# Investigation of PPAR*β*/*δ* within Human Dental Pulp Cells: A Preliminary In Vitro Study

**DOI:** 10.1155/2021/8854921

**Published:** 2021-03-18

**Authors:** Caroline L. de Lima, Bruna R. Amorim, Carine Royer, Augusto P. Resende, Maria F. Borin, Francisco A. R. Neves, Ana Carolina Acevedo

**Affiliations:** ^1^Laboratory of Oral Histopathology, Faculty of Health Sciences, University of Brasilia, University Campus Darcy Ribeiro, Brasília, Brazil; ^2^Laboratory of Molecular Pharmacology, Faculty of Health Sciences, University of Brasilia, University Campus Darcy Ribeiro, Brasília, Brazil

## Abstract

Controlling the inflammatory response to restore tissue homeostasis is a crucial step to maintain tooth vitality after pathogen removal from caries-affected dental tissues. The nuclear peroxisome proliferator-activated receptor beta/delta (PPAR*β*/*δ*) is a ligand-activated transcription factor with emerging anti-inflammatory roles in many cells and tissues. However, its expression and functions are poorly understood in human dental pulp cells (hDPCs). Thus, this study evaluated PPAR*β*/*δ* expression and assessed the anti-inflammatory effects evoked by activation of PPAR*β*/*δ* in lipopolysaccharide- (LPS-) induced hDPCs. Our results showed that hDPCs constitutively expressed PPAR*β*/*δ* mRNA/protein, and treatment with LPS increased *PPARβ/δ* mRNA expression. The selective PPAR*β*/*δ* agonist GW0742 significantly decreased inflammation-related mRNA expression in hDPCs (*IL6*, *IL1β*, *TNFα*, *MMP1*, and *MMP2*) and RAW264.7 cells (*Il6* and *Tnfα*). Further, PPAR*β*/*δ* agonist attenuated MMP2/9 gelatinolytic activity in hDPCs. Previously LPS-conditioned hDPCs increased the migration of RAW264.7 cells through the membrane of a Transwell coculture system. Conversely, pretreatment with GW0742 markedly decreased macrophage recruitment. These findings provide among the first evidence that hDPCs express PPAR*β*/*δ*. In addition, they suggest that activation of PPAR*β*/*δ* by GW0742 can attenuate some cellular and molecular in vitro aspects related to the inflammatory process, pointing out to investigate its potential target role in dental pulp inflammation.

## 1. Introduction

After traumatic injuries and dental caries, a natural defense response takes place within the dentin-pulp complex. When controlled and self-limited, resolving inflammation stimulates regenerative events [1]. These culminate in reactionary dentin production by the primary odontoblasts [2] or, if these cells die, in reparative dentin production by the stem/progenitor cells present in the pulp tissue [3]. The signalling events related to stem cell recruitment and differentiation into a new generation of odontoblast-like cells are complex and not fully understood. However, it is now evident that many molecules, which classically act as inflammatory mediators, including bacterial components, reactive oxygen species (ROS), and cytokines, are also involved in repair response, in a time- and concentration-dependent manner [4–6]. Further, activation of canonically related inflammatory pathways, such as NF*κ*B and MAPK, can also signal in favour of the repair process [7, 8]. Potentially, while relatively low levels of cytokines and growth factors can stimulate repair, high amounts of these molecules, as a result of more intense/persistent bacterial challenges and inflammation, can actively inhibit tertiary dentinogenesis [9]. These findings, together with the tissue breakdown caused by the course of the immune/inflammatory process within an inextensible environment, highlight the need for regenerative approaches based on therapeutic targets to attenuate the inflammation.

Peroxisome proliferator-activated receptor beta/delta (PPAR*β*/*δ*) is a ligand-activated transcription factor that belongs to the nuclear hormone receptor (NR) superfamily. Amongst endogenous ligands are fatty acids, prostaglandins, and leukotrienes, while synthetic agonists include GW0742, a selective high-affinity agonist, widely used in research to explore the role of PPAR*β*/*δ* [10]. Besides major functions in the metabolism, PPAR*β*/*δ* activation displays anti-inflammatory/immune roles, by negatively interfering with proinflammatory transcription factor signalling pathways [11]. Additionally, PPAR*β*/*δ* can act in cell proliferation, differentiation, apoptosis, and angiogenesis, key cellular processes involved in healing and regeneration [12]. Together, such pleotropic functions make PPAR*β*/*δ* a potential therapeutic target to be explored in the dentin-pulp context. However, despite being broadly expressed, this NR has not been reported in dental pulp cells, nor have its related roles. Thus, the aim of this study was to examine whether human dental pulp cells (hDPCs) express PPAR*β*/*δ* and to gain insight into its anti-inflammatory effects.

## 2. Materials and Methods

This study was approved by the Ethics Board of the School of Health Science at the University of Brasilia (number 1400631), and informed consent was obtained from all participants.

### 2.1. Cell Cultures and Treatments

Primary hDPCs were harvested from extracted nonerupted, caries-free third molars with partially root formation of eleven young donors (aged 18 to 21), without systemic disorder, and with no history of regular medication intake. These criteria were considered because the result reproducibility can be affected by the donor tooth conditions, such as stage of development [13] or retained/erupted tooth [14], the donor age [15], and the presence of systemic diseases [16]. Primary cultures were established by the explant outgrowth method [17]. Briefly, immediately after tooth extraction, dental pulps were removed, and the tissues were minced into small fragments and placed into 35 mm culture dishes with high-glucose Dulbecco's modified Eagle medium (DMEM; Sigma-Aldrich, St Louis, MO) supplemented with 20% fetal bovine serum (FBS; Invitrogen, Carlsbad, CA) and antibiotics (50 U/mL penicillin and 50 mg/mL streptomycin; Sigma-Aldrich). The fragments were stabilized with a glass coverslip. Cells were incubated at 37°C in a humidified incubator at 5% CO_2_ and culture medium was replaced every 2-3 days. Confluent cells were subcultured with DMEM supplemented with 10% FBS and antibiotics. All procedures were done in biohazard laminar flow hood and under the sterile conditions, following a rigid laboratory routine with the best practices to avoid misidentification or cross-contamination. Cells at passage 4 were used for all experiments. The RAW264.7 murine macrophage cell line was purchased from the American Type Culture Collection (ATCC® TIB71™, Manassas, VA) and kindly provided by Dr. Paul Webb from the Methodist Research Institute, Houston, TX. RAW264.7 cells were maintained in high-glucose DMEM containing 10% FBS, 50 U/mL penicillin, and 50 *μ*g/mL streptomycin. All experiments were conducted using serum-free medium, with serum starved 24 h before each experiment.

GW0742 (Cayman Chemical, Ann Arbor, MI) was dissolved in dimethyl sulphoxide (DMSO), and cells were pretreated with 0.01, 0.1, and 1.0 *μ*M of GW0742 or with vehicle (DMSO 0.1%). Such concentrations were selected based on the EC50 of 1.1 nM, previously reported in transactivation assays [18], and considering other studies that used this agonist to assess aspects related to inflammation [19, 20]. Because no statistical difference was observed between DMSO 0.1%-treated cells and cells treated with medium only (Supplementary Fig. 1), the effects of GW0742 were expressed in comparison to the vehicle group. For inflammatory stimulus, cells were exposed to lipopolysaccharides (LPS) from *Escherichia coli* 0111:B4 (Sigma-Aldrich). The 2 *μ*g/mL LPS concentration was selected to perform the experiments on hDPC based on a pilot proinflammatory gene expression assay. The diagrams of experimental protocols are detailed in Supplementary Fig. 2.

### 2.2. Immunofluorescence Staining

Cells seeded (2.63 × 10^3^ cells/cm^2^) onto glass coverslips were rinsed with phosphate-buffered saline (PBS), fixed with methanol (10 minutes at room temperature [RT]), and permeabilized/blocked overnight (4°C with humidity) with 0.1% Tween-20/1% bovine serum albumin (BSA)/5% normal goat serum (Reactolab SA, Servion, Switzerland). Then, cells were incubated overnight (4°C with humidity) with rabbit anti-human PPAR*β*/*δ* (1 : 100, sc-7197, Santa Cruz Biotechnology Inc., Dallas, TX). Finally, cells were incubated with secondary antibody Alexa Fluor® 594 (Invitrogen) goat anti-rabbit IgG (1 : 200) for 30 minutes at RT under agitation and protection from light exposure. The cell nuclei were labelled with diamidino-phenyl-indole (DAPI, Invitrogen) for 5 minutes, and the coverslips were mounted onto microscope slides using Fluoromount-G® (SouthernBiotech, Birmingham, AL). Microphotographs were performed using the Axio Imager M2 microscope (Zeiss, Göttingen, Germany).

### 2.3. MTT Cell Viability

hDPCs were seeded (1.56 × 10^4^ cells/cm^2^) in 96-well plates with standard medium. After 24 hours, the medium was replaced with DMEM/2% FBS containing GW0742 (0.01, 0.1, or 1.0 *μ*M) or vehicle for 1 to 6 days, with a medium change every 2 days. Cell viability was assessed by adding 0.5 mg/mL MTT 3-(4,5-dimethylthiazol-2-yl)-2,5-diphenyltetrazolium bromide (Sigma-Aldrich) per well. The formazan crystals produced were solubilized in 200 *μ*L of DMSO, and optical density was measured at a wavelength of 570 nm with a DTX 800 reader (Beckman Coulter, CA).

### 2.4. Quantitative Real-Time Polymerase Chain Reaction

Total RNA was isolated using TRI Reagent (Sigma-Aldrich), followed by DNase I (Sigma-Aldrich) treatment. cDNA was synthesized from 400 ng total RNA by using a High Capacity cDNA Reverse Transcription Kit (Applied Biosystems, Foster City, CA). Quantitative PCR was performed in triplicate in 10 *μ*L reactions by using a PowerUp SYBR® Green Master Mix (Applied Biosystems). Gene and primer sequences are listed in Supplementary Table 1. Relative quantification was calculated using the 2^-*ΔΔ*Ct^ method [21]. For more details, see the Supplementary file (available here).

### 2.5. Gelatin Zymography

Supernatants from treated hDPCs were collected and quantified by using the Qubit® Protein Assay kit (Invitrogen). Samples were mixed with a nonreducing sample buffer (0.05 M Tris-HCL, pH 6.8, 2% sodium dodecyl sulphate [SDS], and 5% glycerol) and electrophoresed (150 V, 4°C) on 8% SDS-polyacrylamide gel with 1% gelatin from porcine skin, type A (Sigma-Aldrich). Afterwards, gels were rinsed twice with 2.5% Triton X-100 for 30 minutes at RT. They were then incubated overnight (37°C) in a reaction buffer (50 mM Tris-HCL, pH 7.4, and 10 mM CaCl_2_), rinsed with distilled water, stained with 0.1% Coomassie blue (PlusOne Coomassie Tablets PhastGel® Blue R350, GE Healthcare, Chicago, IL) in 30% methanol/10% acetic acid solution for 30 minutes, and finally destained with 20% acetic acid solution. Band density was measured using ImageJ software (Rasband Wayne, National Institute of Health, Bethesda, MD). The molecular weight was estimated with Precision Plus Protein® Kaleidoscope® Standards (Bio-Rad, Hercules, CA).

### 2.6. Chemotaxis Assay

Because murine-derived RAW264.7 had previously shown to respond to xenobiotic stimulus from other cells, such as human periodontal ligament stem cells [22–24], human osteogenic sarcoma cells (SaOS-2) [25], U87 human glioma cells [26], and hDPCs [27], this cell model was used in the present study to test the chemotactic effects of hDPCs in a Transwell coculture system (Supplementary Fig. 2B). Briefly, preconditioned hDPCs were put in contact with inserts of a Transwell system (polycarbonate membrane inserts with 6.5 mm diameter and 8 *μ*m pore size; Corning Inc., Corning, NY) containing RAW264.7 cells (1.0 × 10^5^ in serum-free medium). After 14 hours of coculture, nonmigratory RAW264.7 cells in the upper side of the membrane were removed, while transmigrated cells were fixed with methanol for 20 minutes and stained with DAPI (1 : 4000) for 5 minutes. Microphotographs were taken in five different fields (40x) with Axio Observer D1 (Zeiss). The number of migrated cells was measured using ImageJ software.

### 2.7. Statistical Analysis

Normal distribution of data was tested by the Shapiro–Wilk test. Statistical differences among groups were tested by one-way ANOVA and post hoc Newman−Keuls or by Kruskal–Wallis and post hoc Dunn's test. The unpaired 2-tailed Student's *t* test was applied to test for significant differences between two groups. The software GraphPad Prism 5.03 (GraphPad Software, Inc., San Diego, CA) was used for statistical analysis and graphics design. Statistical analyses were performed on the results of at least three different hDPCs performed in duplicates each one. *p* < 0.05 was accepted as statistically significant.

## 3. Results

### 3.1. hDPCs Expressed PPAR*β*/*δ*

**Figure 1:**
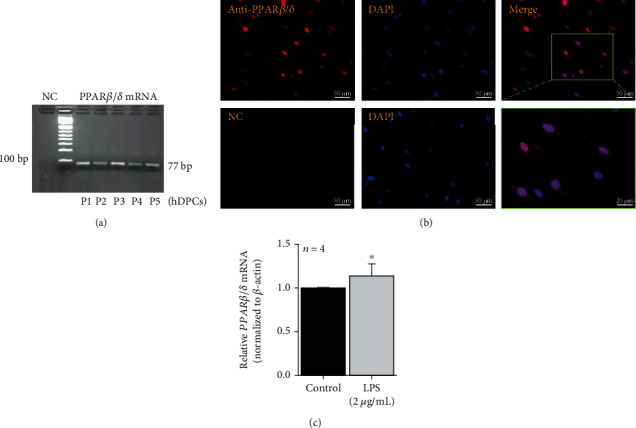
Human dental pulp cells express PPAR*β*/*δ*: (a) *PPARβ/δ* mRNA was ubiquitously expressed in all five hDPC cultures; (b) representative immunofluorescence labeling of hDPCs stained with anti-PPAR*β*/*δ* antibody (red) and nucleus (blue/DAPI). Merged images demonstrated that PPAR*β*/*δ* was widely immunolocalized in the nuclei. NC: negative control, without primary anti-PPAR*β*/*δ* antibody. (c) Treatment with 2 *μ*g/mL LPS increased *PPARβ/δ* mRNA level (^∗^*p* < 0.05 vs. control by Mann-Whitney test; mean ± S.E.M.*n* = per group). Control = DMSO 0.1%.

*PPARβ/δ* mRNA was expressed by all five primary hDPC cultures assessed (average *PPARβ/δ* Ct, min–max: 22.07, 21.79–22.32/average *β-*actin Ct, min–max: 14.37, 13.69–14.99). Primers detected a single band with the appropriate size (77 bp), consistent with the predicted amplicon (Figure 1(a)). The immunofluorescence assay confirmed PPAR*β*/*δ* protein expression and revealed a primary significant nuclear localization (Figure 1(b)).

### 3.2. *PPARβ/δ* Gene Expression Was Upregulated in LPS-Stimulated hDPCs

Treatment with 2 *μ*g/mL LPS slightly but significantly increased the *PPARβ/δ* level (*p* < 0.05) (Figure 1(c)). A similar but more pronounced increase was obtained after the hydrogen peroxide (H_2_O_2_) stimulus (Supplementary Fig. 3A). This compound was used to mimic a more pronounced inflammatory process as the carious lesion progresses, with increased levels of ROS and cytokines [28].

### 3.3. PPAR*β*/*δ* Agonist Attenuated Inflammatory Gene Expression

**Figure 2:**
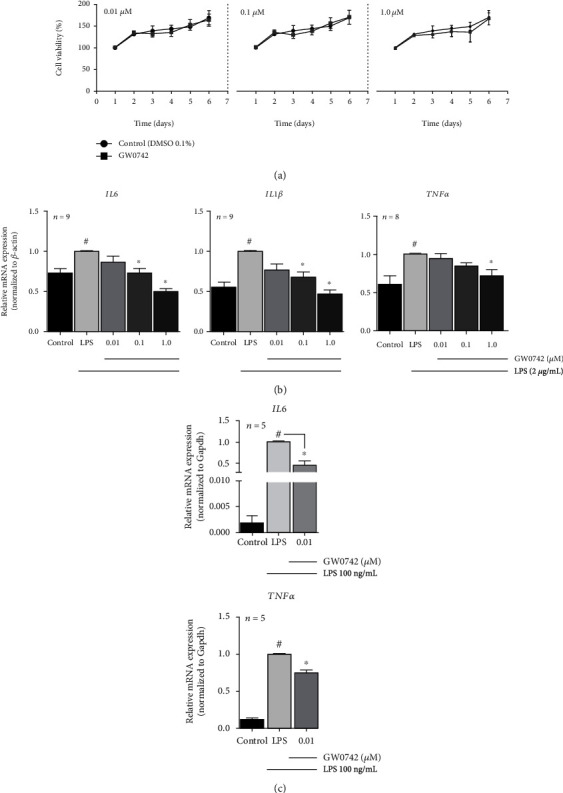
GW0742-activated PPAR*β*/*δ* represses cytokine gene expression in LPS-stimulated hDPCs: (a) treatment with GW0742 (0.01, 0.1, and 1.0 *μ*M) did not alter hDPC viability. (b) Real-time qPCR analyses of *IL6*, *IL1β*, and *TNFα* in cultured hDPCs pretreated with GW0742 (for 24 h) and added LPS (2 *μ*g/mL) for 4 h before harvesting. GW0742 significantly reduced LPS-induced *IL6* and *IL1β* at 0.1 *μ*M and LPS-induced *IL6*, *IL1β*, and *TNFα* at 1.0 *μ*M. (c) Pretreatment with 0.01 *μ*M GW0742 (for 24 h) significantly reduced *Il6* and *Tnfα* mRNA levels in LPS-stimulated RAW264.7 cells (for 24 h) (*p* < 0.05; ^#^vs. control; ^∗^vs. LPS by Kruskal–Wallis and post hoc Dunn's test; mean ± S.E.M.*n* = per group). Control = DMSO 0.1%.

The PPAR*β*/*δ* agonist GW0742 was first tested for cytotoxicity, and data showed that none of the concentrations used affected cell viability at any time-point considered (Figure 2(a)). We also tested the effect of LPS alone or in association with GW0742 in cell viability, and we did not find any statistical difference between vehicle-treated cells and cells treated with LPS alone or with GW0742 (Supplementary Fig. 4). Then, we assessed *IL6*, *IL1β*, and *TNFα* expression. As expected, exposure of hDPCs to 2 *μ*g/mL LPS increased inflammatory gene expression. Conversely, GW0742 pretreatment significantly reduced LPS-induced *IL6* and *IL1β* at 0.1 *μ*M, and LPS-induced *IL6*, *IL1β*, and *TNFα* at 1.0 *μ*M concentration (Figure 2(b)). GW0742 repressing proinflammatory gene expression was also observed with H_2_O_2_ stimulus (Supplementary Fig. 3B). The results with RAW264.7 cells showed that the pretreatment with GW0742 (0.01 *μ*M) significantly reduced *Il6* and *Tnfα* levels in LPS-stimulated cells, compared with the group exposure to LPS alone (Figure 2(c)).

### 3.4. PPAR*β*/*δ* Agonist Attenuated *MMP* Expression and Gelatinolytic Activities

**Figure 3:**
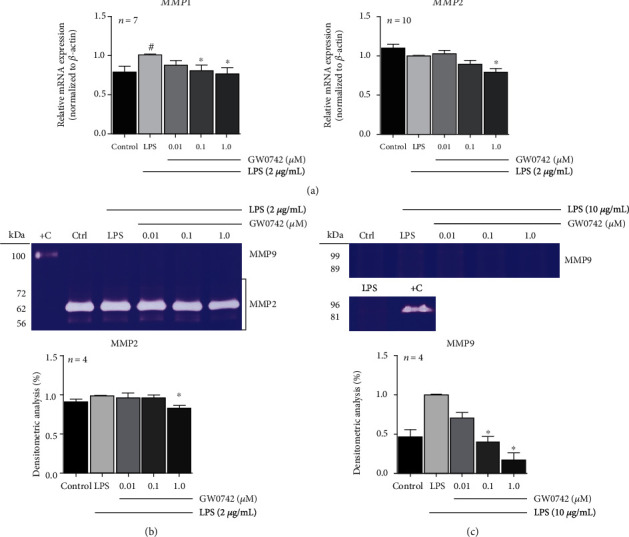
GW0742-activated PPAR*β*/*δ* represses *MMP* gene expression and gelatinolytic activity: (a) pretreatment with GW0742 repressed *MMP1* and *MMP2* gene expression and gelatinolytic activity of (b) MMP2 and (c) MMP9 in LSP-stimulated hDPCs (*p* < 0.05; ^#^vs. control; ^∗^vs. LPS by Kruskal–Wallis and post hoc Dunn's test; mean ± S.E.M.*n* = per group). Control = DMSO 0.1%. +C = positive control, supernatant from macrophage RAW264.7 cells.

After exposing cells to 2 *μ*g/mL LPS, data revealed an increase in *MMP1* level, with no impact in *MMP2* expression. When hDPCs were pretreated with GW0742, a significant downregulation of *MMP1* and *MMP2* levels at 1.0 *μ*M concentration was observed (Figure 3(a)). Similar results were found with H_2_O_2_ stimulus (Supplementary Fig. 3C). In accordance with gene expression data, LPS stimulation did not affect MMP2 proteolytic activity, but pretreatment with 1.0 *μ*M GW0742 caused a slight reduction on the gelatinolytic activity that was statistically significant (Figure 3(b)). In these conditions, we did not detect the activity of MMP9. However, when hDPCs were treated with a higher dose of LPS (10 *μ*g/mL), a slim proteolytic band of MMP9 was observed. Densitometric analysis showed a slight enhancement in MMP9 activity after exposure cells to LPS, and a dose-dependent decrease in the proteolytic band when cells were pretreated with GW0742 (Figure 3(c)). To confirm them to be MMPs with gelatinolytic activity, an EDTA inhibition assay was performed (data are available on request), excluding other MMPs with low specific activity for gelatin [29].

### 3.5. hDPCs Previously Conditioned with GW0742 Suppressed Macrophage Recruitment

**Figure 4:**
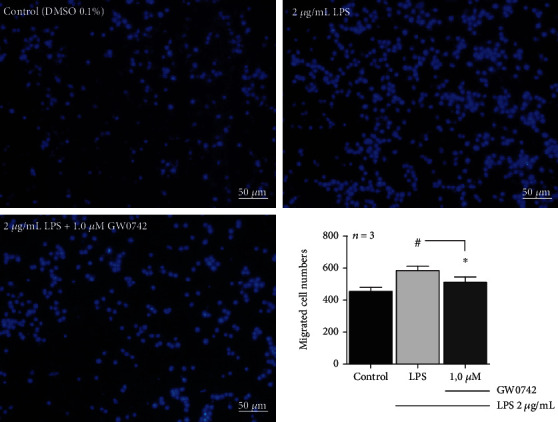
Pretreatment with GW0742 downregulates LPS-stimulated hDPCs' chemotactic ability: LPS-stimulated hDPCs recruited more RAW264.7 macrophage cells when compared with control cells. Pretreatment of LPS-stimulated hDPCs with 1.0 *μ*M GW0742 significantly reduced the number of migrated macrophage cells (*p* < 0.05; ^#^vs. control; ^∗^vs. LPS by one-way ANOVA and post hoc Newman−Keuls; mean ± S.E.M.*n* = per group).

hDPCs previously conditioned with 2 *μ*g/mL LPS recruited more macrophage cells, compared with control cells (DMSO 0.1%). Conversely, when LPS-stimulated hDPCs were pretreated with 1.0 *μ*M GW0742, the number of recruited RAW264.7 cells through the Transwell membrane significantly reduced (Figure 4).

## 4. Discussion

In this study, we provide among the first evidence of PPAR*β*/*δ* mRNA and protein expression in dental pulp cells. We also revealed that PPAR*β*/*δ* activation by the specific ligand GW0742 improved the inflammatory profile by attenuating some aspects related with the inflammatory process, including proinflammatory cytokine gene expression, MMP gene expression, gelatinase activity, and macrophage recruitment.

To explore whether PPAR*β*/*δ* is expressed by hDPCs, we firstly screened the mRNA expression by using real-time qPCR and confirmed the protein expression and cell localization by immunofluorescence. The basal PPAR*β*/*δ* localization was predominantly on the nucleus of hDPCs, in agreement with other reports [30, 31]. Like many NRs, PPAR*β*/*δ* is generally localized on the nucleus, binding to the promoter regions of its target genes as a heterodimer with a retinoid X receptor. Canonically, in the absence of agonists, PPAR*β*/*δ* mediates gene repression, while gene expression is induced in the presence of its agonists [32]. In addition, activated PPAR*β*/*δ* can also repress genes independently of DNA binding, by interacting with transcription factors, for example, [33]. Thus, its localization on the nucleus is compatible with the mode of action and suggests that PPAR*β*/*δ* might exert some roles in hDPCs. Indeed, when hDPCs were stimulated with LPS, a component of Gram-negative bacteria that triggers the innate immune response [34, 35], *PPARβ/δ* expression significantly increased. Such an increase was also observed after stimulus with H_2_O_2_, suggesting a pathophysiologic role of this NR.

To gain insights into PPAR*β*/*δ* anti-inflammatory function, we pretreated LPS-stimulated cells with three noncytotoxic concentrations of GW0742, and then, the *IL6*, *IL1β*, and *TNFα* mRNA expression was assessed. In our study, treatment with GW0742 significantly reduced proinflammatory cytokine gene expression in hDPCs, in agreement with other reports that ascribe to PPAR*β*/*δ* a regulatory role on transcription and inflammatory mediator production [19, 36–39]. The potential of GW0742 in repressing proinflammatory gene expression in hDPCs was also supported by a second inflammatory *in vitro* model with H_2_O_2_. Because macrophages seem to play a critical role in the progression of pulpal inflammation, we next tested whether PPAR*β*/*δ* agonist could modulate cytokine gene expression in LPS-stimulated RAW264.7 cell line, and similar to dental pulp cells, a reduction in *Il6* and *Tnfα* mRNA levels was also observed. The mechanisms by which the PPAR*β*/*δ* ligand reduces inflammatory response in hDPCs should be assessed. However, data from macrophages and other cell lines suggested a direct inhibition of NF*κ*B and STAT transactivation by activated PPAR*β*/*δ* without direct DNA contact [19, 37]. The association with the transcriptional repressor protein B cell lymphoma 6 (BCL6) has also been described: unliganded PPAR*β*/*δ* can physically associate with BCL6, thus preventing BCL6 to repress proinflammatory genes. Conversely, in the presence of the agonist, PPAR*β*/*δ* dissociates from BCL6, releasing it to suppress proinflammatory pathways [38, 40]. Canonical direct transcriptional induction of anti-inflammatory genes, such as TGF*β* [41] and antioxidative genes [38, 39], might be the other way activated PPAR*β*/*δ* exerts its actions.

Because pulp tissue destruction involves extracellular matrix breakdown by the action of proteolytic enzymes, such as matrix metalloproteinases, released to facilitate immune cells recruitment [28], the effects of GW0742 in MMP gene expression and gelatinase activity were also investigated. In our study, pretreatment with 1.0 *μ*M GW0742 significantly reduced the expression of the collagenase *MMP1* and the gelatinase *MMP2*. A reduction in MMP2 proteolytic activity was also observed. Interestingly, LPS did not increase MMP2 gene expression and activity. We attributed these results to the posttreatment period evaluated (24 h) [42]. The MMP9 levels were relatively low, with undetectable basal transcript levels and protein activity for most cultures investigated. However, when we increased LPS concentration, an MMP9 proteolytic activity was observed. Additionally, pretreatment with GW0742 effectively decreased its activity.

Finally, to further explore the biologic relevance of the PPAR*β*/*δ* activation, hDPCs previously treated with GW0742/LPS were tested for their chemotactic effect on macrophage recruitment. Among immune cells, macrophages likely predominate in health and inflamed pulp tissue [43]. Further, their number increases with the progression of dental caries, playing a key role in the course of pulp inflammation and necrosis [44]. Thus, a tight regulation of macrophage recruitment might protect the pulp from excessive inflammation and collateral damage. Here, our study revealed that pretreated LPS-stimulated hDPCs with GW0742 markedly altered their chemotactic gradient, resulting in suppression of RAW264.7 migration, with likely phenotypic alteration, as recently suggested [27]. Indeed, LPS-stimulated RAW264.7 cells cocultured with hDPCs expressed less proinflammatory factors compared to LPS-stimulated ones. Also, RAW cells cocultured with DPSCs appeared more morphologically elongated than cells cultured without hDPCs, which seem clustered and round-shaped [27].

Taken together, our findings indicate that GW0742 can contribute to attenuate the proinflammatory environment in the pulp, protecting it from excessive inflammation and destructive damage. The anti-inflammatory properties together with repair induction might further increase the therapeutic potential of activated PPAR*β*/*δ* in the dentin-pulp complex. Our preliminary results from a pilot assay showed an increase in nodule formation by GW0742-treated hDPCs (Supplementary Fig. 5). A previous study showed that ligand-activated PPAR*β*/*δ* induced osteogenic differentiation of osteoblasts, with an increase in bone nodule formation and alkaline phosphatase expression. They suggested that PPAR*β*/*δ* activation can amplify Wnt-dependent and *β*-catenin-dependent signalling through transcriptional regulation of the low-density lipoprotein receptor-related protein 5 (LRP5) and direct interaction with *β*-catenin [45]. Due to the importance of the Wnt/*β*-catenin signalling pathway in dentin formation and repair, its interaction with PPAR*β*/*δ* might be a new field to investigate.

The present study has several limitations. The preventive design of experiments, despite being widely used in agonism studies, does not match with clinical reality. The sample size within some experiments is another limitation, and further studies are suggested to endorse reproducibility. Furthermore, although we have shown promising gene expression results, it is also important to investigate whether such effects are reproducible in the protein levels. Thus, future protein detection experiments are necessary to better support the present results. Also, further experiments are needed to elucidate the effects of GW0742 itself in gene expression, to investigate the signalling pathway involved with the anti-inflammatory activity of activated PPAR*β*/*δ*, to explore how activated PPAR*β*/*δ* alters hDPC chemotaxis, and to confirm whether the effects are receptor-dependent or independent. Nevertheless, our findings highlight a new target to be explored in future research and open potential new therapeutic avenues for the treatment of pulp diseases, which could be used in association with microbial control.

## 5. Conclusion

In conclusion, this study is the first to demonstrate PPAR*β*/*δ* expression in human dental pulp cells, and it suggested that activated PPAR*β*/*δ* has anti-inflammatory effects in hDPCs by preventing proinflammatory and *MMP* gene expression, suppressing gelatinase activity and macrophage recruitment.

## Data Availability

Data used to support the findings of this study are available from the corresponding author upon request.

## Supplementary Materials

Supplementary Materials Supplementary information may be found in the online version of this article: Supplementary methods. Quantitative real-time polymerase chain reaction: all quantitative real-time PCR assays were performed on the Applied Biosystems StepOnePlus™ Real-Time PCR Systems, and the data were generated by StepOne v2.1 Software. The cycle conditions were 50°C for 2 minutes, 95°C for 10 minutes, followed by 40 cycles at 95°C for 15 s and 60°C for 1 minute. Samples were normalized to beta-actin (hDPCs) or to glyceraldehyde 3-phosphate dehydrogenase enzyme (*Gapdh*; RAW264.7 cells). To assure specificity, a melt curve was obtained for all qPCR products. Further, standard curves were obtained for each primer pair to assess the efficiency of amplification. Mineralization assay: confluent hDPCs were cultured in mineralization medium (alpha minimum essential medium (*α*-MEM) with 10% FBS, antibiotics, 10 mmol/L *β*-glycerophosphate, 10 nmol/L dexamethasone, and 50 *μ*g/mL ascorbic acid) containing GW0742 (1.0 *μ*M) or vehicle (DMSO 0.01%). After 28 days, cells were rinsed with PBS, fixed with ethanol for 30 minutes at RT, and stained for 10 minutes with 2% Alizarin red S (Sigma-Aldrich) solution, pH 4.2, at RT. Cells were then rinsed 3 times with distilled water to reduce nonspecific staining. This experiment was performed in triplicate. Supplementary Table 1 Gene and primer sequences. Supplementary Figure 1 MTT cell viability assay to assess DMSO safety. hDPCs were incubated with DMEM/2% FBS containing DMSO 0.1% or left untreated (hDPCs), and metabolic activity was evaluated daily during 6 days by MTT colorimetric assay. Supplementary Figure 2 Diagrams of experimental protocol for GW0742 treatment followed by inflammatory stimulus with LPS in hDPCs (2 *μ*g/mL) and RAW264.7 cells (100 ng/mL), for (a) gene expression and gelatinolytic activity, and for (b) chemotaxis assay using coculture Transwell system. Supplementary Figure 3 (a) Treatment with 300 *μ*M H_2_O_2_ significantly increases *PPARβ/δ* mRNA level; pretreatment with GW0742 repressed (b) *IL6*, *IL1β*, and *TNFα* and (c) *MMP1* and *MMP2* gene expression in H_2_O_2_-stimulated hDPCs (*p* < 0.05; ^#^vs. control; ^∗^vs. H_2_O_2_; mean ± S.E.M.). Supplementary Figure 4 MTT cell viability assay to assess LPS and GW0742 safety. hDPCs were incubated with DMEM/2% FBS containing DMSO 0.1% (control group) or LPS (0.1 *μ*g/mL or 10 *μ*g/mL) alone or in association with three concentrations of GW0742 (0.01 *μ*M, 0.1 *μ*M, or 1.0 *μ*M), and metabolic activity was evaluated daily for 6 days by MTT colorimetric assay. (a) Treatment with 0.1 *μ*g/mL or 10 *μ*g/mL LPS did not affect cell viability at any time point considered (*p* > 0.05 by one-way ANOVA and post hoc Newman−Keuls). (b, c) Treatment with 0.1 *μ*g/mL or 10 *μ*g/mL LPS in association with GW0742 did not impair cell viability at any time point considered (*p* > 0.05 by one-way ANOVA and post hoc Newman−Keuls). *n* = 9. Supplementary Figure 5 GW0742-activated PPAR*β*/*δ* increases calcified nodule formation. Preliminary (*n* = 1) Alizarin red staining of hDPCs after 28 days of osteo/odontogenesis differentiation in the presence of the indicated concentrations of GW0742 or the vehicle (DMSO 0.01%). Mineralized medium: *α*MEM with 10% FBS, 10 mM *β*-glycerophosphate, 10 nM dexamethasone, and 50 *μ*g/mL ascorbic acid.
