# Supplementary material for: Investigation of PPARβ/δ within Human Dental Pulp Cells: A Preliminary In Vitro Study
Source: PPAR Res. 2021 Mar 18;2021:8854921. doi: 10.1155/2021/8854921 (PMC7997762; doi:10.1155/2021/8854921)
Supplement: Supplementary Materials — Supplementary information may be found in the online version of this article: Supplementary methods. Quantitative real-time polymerase chain reaction: all quantitative real-time PCR assays were performed on the Applied Biosystems StepOnePlus™ Real-Time PCR Systems, and the data were generated by StepOne v2.1 Software. The cycle conditions were 50°C for 2 minutes, 95°C for 10 minutes, followed by 40 cycles at 95°C for 15 s and 60°C for 1 minute. Samples were normalized to beta-actin (hDPCs) or to glyceraldehyde 3-phosphate dehydrogenase enzyme (Gapdh; RAW264.7 cells). To assure specificity, a melt curve was obtained for all qPCR products. Further, standard curves were obtained for each primer pair to assess the efficiency of amplification. Mineralization assay: confluent hDPCs were cultured in mineralization medium (alpha minimum essential medium (α-MEM) with 10% FBS, antibiotics, 10 mmol/L β-glycerophosphate, 10 nmol/L dexamethasone, and 50 μg/mL ascorbic acid) containing GW0742 (1.0 μM) or vehicle (DMSO 0.01%). After 28 days, cells were rinsed with PBS, fixed with ethanol for 30 minutes at RT, and stained for 10 minutes with 2% Alizarin red S (Sigma-Aldrich) solution, pH 4.2, at RT. Cells were then rinsed 3 times with distilled water to reduce nonspecific staining. This experiment was performed in triplicate. Supplementary Table 1 Gene and primer sequences. Supplementary Figure 1 MTT cell viability assay to assess DMSO safety. hDPCs were incubated with DMEM/2% FBS containing DMSO 0.1% or left untreated (hDPCs), and metabolic activity was evaluated daily during 6 days by MTT colorimetric assay. Supplementary Figure 2 Diagrams of experimental protocol for GW0742 treatment followed by inflammatory stimulus with LPS in hDPCs (2 μg/mL) and RAW264.7 cells (100 ng/mL), for (a) gene expression and gelatinolytic activity, and for (b) chemotaxis assay using coculture Transwell system. Supplementary Figure 3 (a) Treatment with 300 μM H2O2 significantly increases PPARβ/δ mRNA [file 8854921.f1.zip › Supplementary file_final (1).docx]

**Supplementary methods**

Quantitative real-time polymerase chain reaction

All quantitative real-time PCR assays were performed on the Applied Biosystems StepOnePlus ™ Real-Time PCR Systems and the data were generated by StepOne v2.1 Software. The cycle conditions were 50°C for 2 minutes, 95°C for 10 minutes, followed by 40 cycles at 95°C for 15 s and 60°C for 1 minutes. Samples were normalized to beta-actin (hDPCs) or to glyceraldehyde 3-phosphate dehydrogenase enzyme (*Gapdh*; RAW 264.7 cells). To assure specificity, a melt curve was obtained for all qPCR products. Further, standard curves were obtained for each primer pair to assess the efficiency of amplification.

Mineralization assay

Confluent hDPCs were cultured in mineralization medium (alpha minimum essential medium (α-MEM) with 10% FBS, antibiotics, and 10 mmol/L β-glycerophosphate, 10 nmol/L dexamethasone, and 50 μg/mL ascorbic acid) containing GW0742 (1,0 μM) or vehicle (DMSO 0.01%). After 28 days, cells were rinsed with PBS, fixed with ethanol for 30 minutes at RT, and stained for 10 minutes with 2% Alizarin red S (Sigma-Aldrich) solution, pH 4.2, at RT. Cells were then rinsed 3 times with distilled water to reduce non-specific staining. This experiment was performed in triplicate.

**Supplementary Table 1.** Gene and primers sequences.

| Gene | Sequence (5’ – 3’) | Amplicon (bp) |
| --- | --- | --- |
| *Homo Sapiens* |  |  |
| *IL6* | F: CCTGAACCTTCCAAAGATGGC  R: TTCACCAGGCAAGTCTCCTCA | 75 |
| *IL1β* | F: ATGATGGCTTATTACAGTGGCAA  R: GTCGGAGATTCGTAGCTGGA | 132 |
| *TNFα* | F: CTGCCCAGACTAGGCAA  R: GGAGAAGGGGTGACCGACT | 71 |
| *MMP1* | F: GGGGTATCCGTGTAGCACAT  R: CAGAGATGAAGTCCGGTTTTTC | 76 |
| *MMP2* | F: GCTACGATGGAGGCGCTAATG  R: TCAGGTATTGCACTGCCAACTC | 169 |
| *PPARβ/δ* | F: ACTGAGTTCGCCAAGAGCATC  R: ACGCCATACTTGAGAAGGGTAA | 77 |
| *β-ACTIN* | F: TCACCCACACTGTGCCCATCTACG  R: CAGCGGAACCGCTCATTGCCAATG | 295 |
| Mus musculus |  |  |
| *Il6* | F: TAGTCCTTCCTACCCCAATTTCC  R: TTGGTCCTTAGCCACTCCTTC | 76 |
| *Tnfα* | F: CCCTCACACTCAGATCATCTTCT  R: GCTACGACGTGGGCTACAG | 61 |
| *Gapdh* | F: AAGGGCTCATGACCACAGTC  R: CAGGGATGATGTTCTGGGCA | 111 |

F, Forward; R, reverse; bp, base pairs.

**Supplementary Figure 1.** MTT cell viability assay to assess DMSO safety. hDPCs were incubated with DMEM/2% FBS containing DMSO 0.1%, or left untreated (hDPCs), and metabolic activity was evaluated daily during 6 days by MTT colorimetric assay.

**Supplementary Figure 2.** Diagrams of experimental protocol for GW0742 treatment followed by inflammatory stimulus with LPS in hDPCs (2 μg/mL) and RAW 264.7 cells (100 ng/mL), for (a) gene expression and gelatinolytic activity, and for (b) chemotaxis assay using co-culture transwell system.

**Supplementary Figure 3.** (a) Treatment with 300 μM H_2_O_2_ significantly increases *PPARβ/δ* mRNA level; Pretreatment with GW0742 repressed (b) *IL6*, *IL1β* and *TNFα*, and (c) *MMP1* and *MMP2* gene expression in H_2_O_2_-stimulated hDPCs (p<0.05; # *vs* control; * *vs* H_2_O_2_. Mean ± S.E.M.).

**Supplementary Figure 4.** MTT cell viability assay to assess LPS and GW0742 safety. hDPC were incubated with DMEN/2% FBS containing DMSO 0.1% (control group) or LPS (0.1 μg/mL or 10 μg/mL) alone or in association with three concentrations of GW0742 (0.01 μM, 0.1 μM or 1.0 μM), and metabolic activity was evaluated daily during 6 days by MTT colorimetric assay. (a) Treatment with 0.1μg/mL or 10 μg/mL LPS did not affect cell viability at any time point considered (P>0.05 by one-way ANOVA and post hoc Newman−Keuls). (b) and (c) Treatment with 0.1μg/mL or 10 μg/mL LPS in association with GW0742 did not impair cell viability at any time point considered (P>0.05 by one-way ANOVA and post hoc Newman−Keuls). n= 9.

**Supplementary Figure 5.** GW0742-activated PPARβ/δ increases calcified nodules formation. Preliminary (n=1) alizarin red staining of hDPCs after 28 days of osteo/odontogenesis differentiation in the presence of the indicated concentrations of GW0742 or the vehicle (DMSO 0.01%). Mineralized medium: αMEM with 10% FBS, and 10 mM β-glycerophosphate, 10 nM dexamethasone, and 50 μg/mL ascorbic acid.
