# Supplementary figures and images for: Investigation of PPARβ/δ within Human Dental Pulp Cells: A Preliminary In Vitro Study
Source: PPAR Res. 2021 Mar 18;2021:8854921. doi: 10.1155/2021/8854921 (PMC7997762; doi:10.1155/2021/8854921)

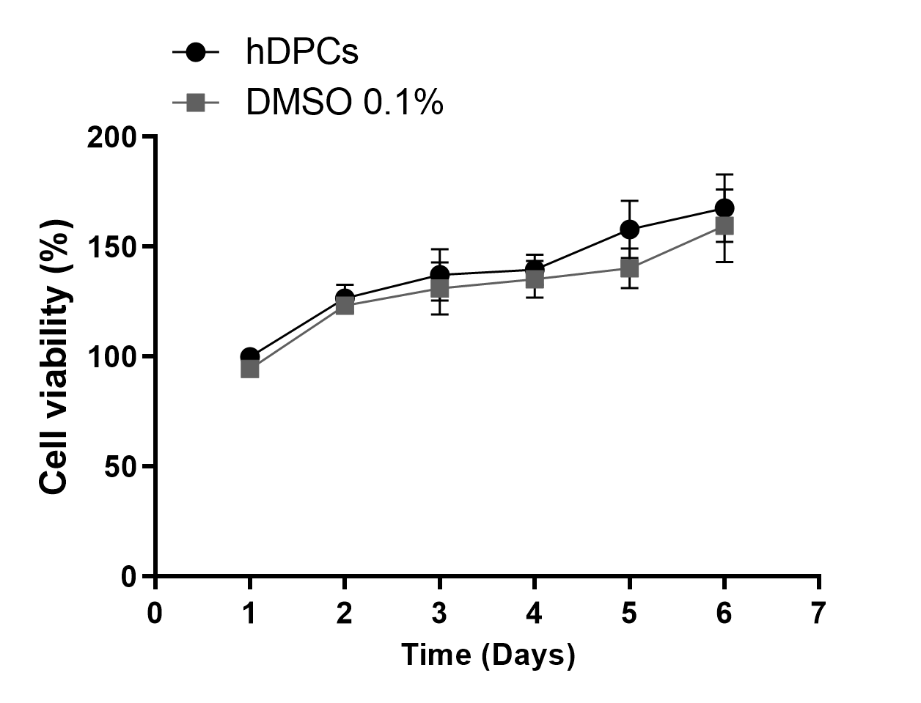

Supplement: Supplementary Materials — Supplementary information may be found in the online version of this article: Supplementary methods. Quantitative real-time polymerase chain reaction: all quantitative real-time PCR assays were performed on the Applied Biosystems StepOnePlus™ Real-Time PCR Systems, and the data were generated by StepOne v2.1 Software. The cycle conditions were 50°C for 2 minutes, 95°C for 10 minutes, followed by 40 cycles at 95°C for 15 s and 60°C for 1 minute. Samples were normalized to beta-actin (hDPCs) or to glyceraldehyde 3-phosphate dehydrogenase enzyme (Gapdh; RAW264.7 cells). To assure specificity, a melt curve was obtained for all qPCR products. Further, standard curves were obtained for each primer pair to assess the efficiency of amplification. Mineralization assay: confluent hDPCs were cultured in mineralization medium (alpha minimum essential medium (α-MEM) with 10% FBS, antibiotics, 10 mmol/L β-glycerophosphate, 10 nmol/L dexamethasone, and 50 μg/mL ascorbic acid) containing GW0742 (1.0 μM) or vehicle (DMSO 0.01%). After 28 days, cells were rinsed with PBS, fixed with ethanol for 30 minutes at RT, and stained for 10 minutes with 2% Alizarin red S (Sigma-Aldrich) solution, pH 4.2, at RT. Cells were then rinsed 3 times with distilled water to reduce nonspecific staining. This experiment was performed in triplicate. Supplementary Table 1 Gene and primer sequences. Supplementary Figure 1 MTT cell viability assay to assess DMSO safety. hDPCs were incubated with DMEM/2% FBS containing DMSO 0.1% or left untreated (hDPCs), and metabolic activity was evaluated daily during 6 days by MTT colorimetric assay. Supplementary Figure 2 Diagrams of experimental protocol for GW0742 treatment followed by inflammatory stimulus with LPS in hDPCs (2 μg/mL) and RAW264.7 cells (100 ng/mL), for (a) gene expression and gelatinolytic activity, and for (b) chemotaxis assay using coculture Transwell system. Supplementary Figure 3 (a) Treatment with 300 μM H2O2 significantly increases PPARβ/δ mRNA [file 8854921.f1.zip › Supp_Figure1.docx]

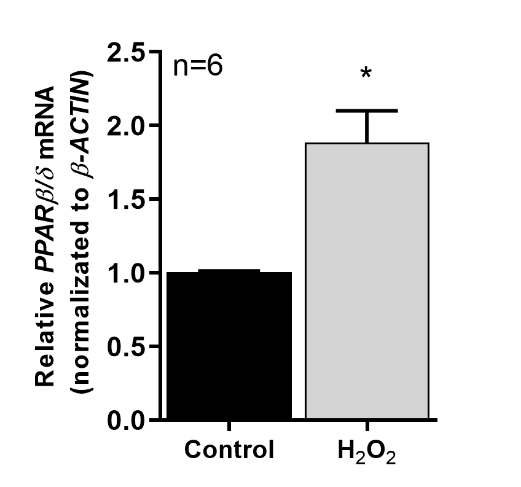

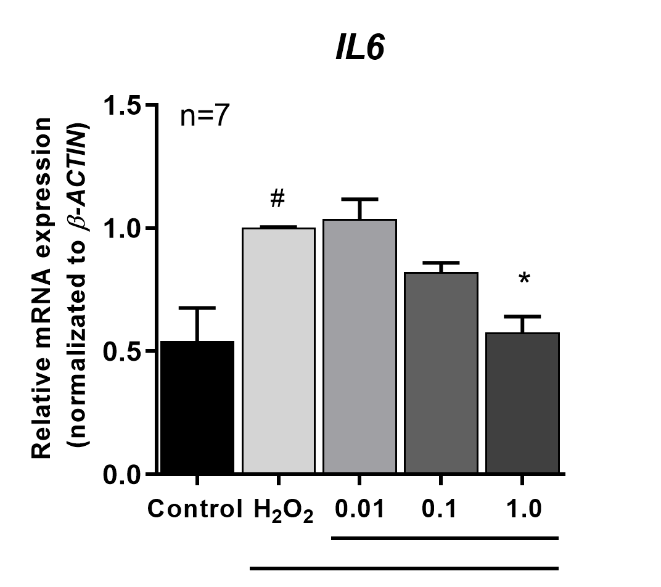

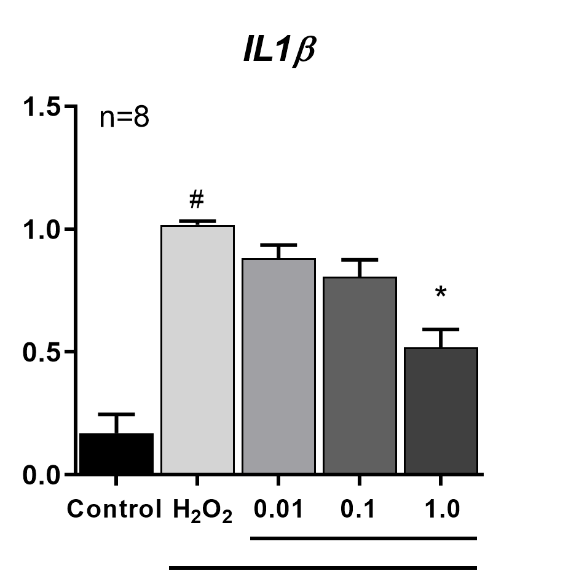

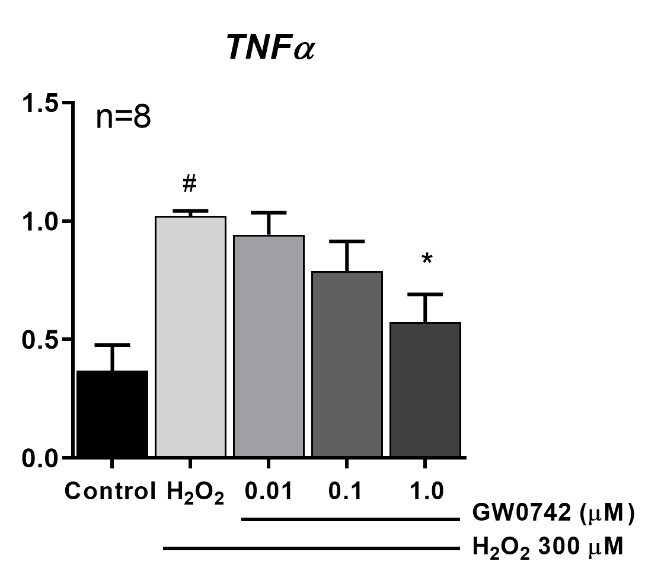


**b**

**a**

**c**

Supplement: Supplementary Materials — Supplementary information may be found in the online version of this article: Supplementary methods. Quantitative real-time polymerase chain reaction: all quantitative real-time PCR assays were performed on the Applied Biosystems StepOnePlus™ Real-Time PCR Systems, and the data were generated by StepOne v2.1 Software. The cycle conditions were 50°C for 2 minutes, 95°C for 10 minutes, followed by 40 cycles at 95°C for 15 s and 60°C for 1 minute. Samples were normalized to beta-actin (hDPCs) or to glyceraldehyde 3-phosphate dehydrogenase enzyme (Gapdh; RAW264.7 cells). To assure specificity, a melt curve was obtained for all qPCR products. Further, standard curves were obtained for each primer pair to assess the efficiency of amplification. Mineralization assay: confluent hDPCs were cultured in mineralization medium (alpha minimum essential medium (α-MEM) with 10% FBS, antibiotics, 10 mmol/L β-glycerophosphate, 10 nmol/L dexamethasone, and 50 μg/mL ascorbic acid) containing GW0742 (1.0 μM) or vehicle (DMSO 0.01%). After 28 days, cells were rinsed with PBS, fixed with ethanol for 30 minutes at RT, and stained for 10 minutes with 2% Alizarin red S (Sigma-Aldrich) solution, pH 4.2, at RT. Cells were then rinsed 3 times with distilled water to reduce nonspecific staining. This experiment was performed in triplicate. Supplementary Table 1 Gene and primer sequences. Supplementary Figure 1 MTT cell viability assay to assess DMSO safety. hDPCs were incubated with DMEM/2% FBS containing DMSO 0.1% or left untreated (hDPCs), and metabolic activity was evaluated daily during 6 days by MTT colorimetric assay. Supplementary Figure 2 Diagrams of experimental protocol for GW0742 treatment followed by inflammatory stimulus with LPS in hDPCs (2 μg/mL) and RAW264.7 cells (100 ng/mL), for (a) gene expression and gelatinolytic activity, and for (b) chemotaxis assay using coculture Transwell system. Supplementary Figure 3 (a) Treatment with 300 μM H2O2 significantly increases PPARβ/δ mRNA [file 8854921.f1.zip › Supp_Figure3.docx]

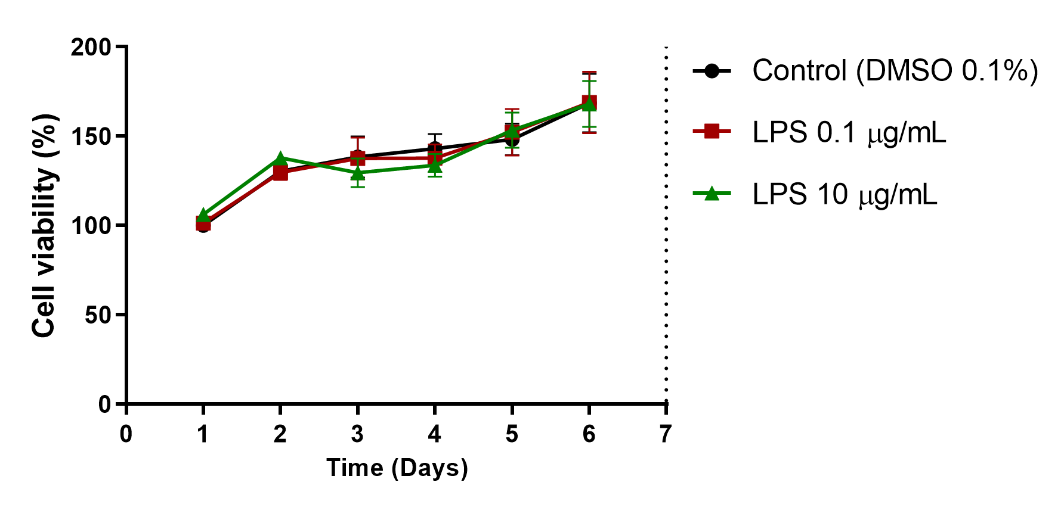

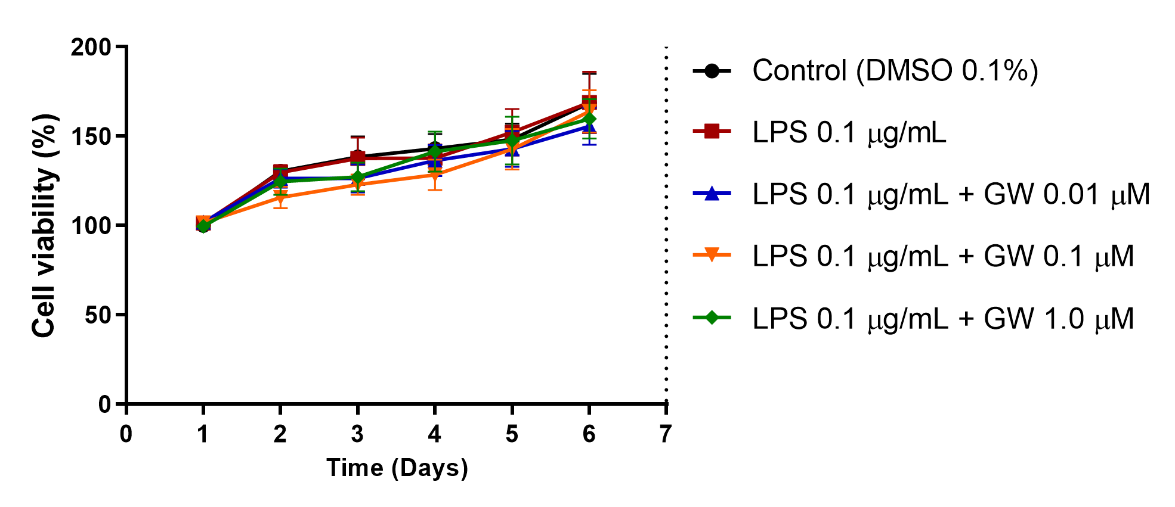

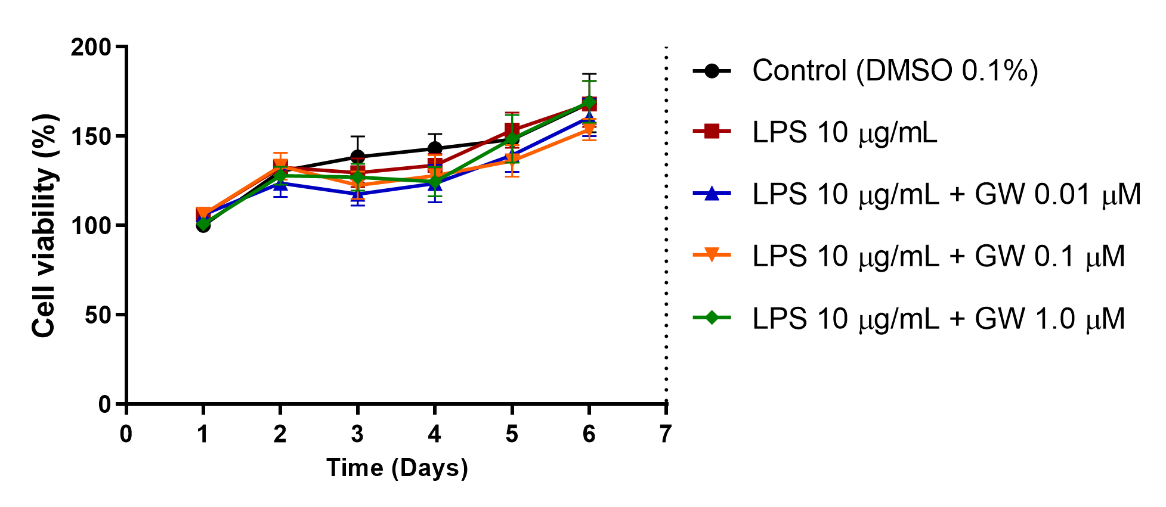


**a**

**b**

**c**

Supplement: Supplementary Materials — Supplementary information may be found in the online version of this article: Supplementary methods. Quantitative real-time polymerase chain reaction: all quantitative real-time PCR assays were performed on the Applied Biosystems StepOnePlus™ Real-Time PCR Systems, and the data were generated by StepOne v2.1 Software. The cycle conditions were 50°C for 2 minutes, 95°C for 10 minutes, followed by 40 cycles at 95°C for 15 s and 60°C for 1 minute. Samples were normalized to beta-actin (hDPCs) or to glyceraldehyde 3-phosphate dehydrogenase enzyme (Gapdh; RAW264.7 cells). To assure specificity, a melt curve was obtained for all qPCR products. Further, standard curves were obtained for each primer pair to assess the efficiency of amplification. Mineralization assay: confluent hDPCs were cultured in mineralization medium (alpha minimum essential medium (α-MEM) with 10% FBS, antibiotics, 10 mmol/L β-glycerophosphate, 10 nmol/L dexamethasone, and 50 μg/mL ascorbic acid) containing GW0742 (1.0 μM) or vehicle (DMSO 0.01%). After 28 days, cells were rinsed with PBS, fixed with ethanol for 30 minutes at RT, and stained for 10 minutes with 2% Alizarin red S (Sigma-Aldrich) solution, pH 4.2, at RT. Cells were then rinsed 3 times with distilled water to reduce nonspecific staining. This experiment was performed in triplicate. Supplementary Table 1 Gene and primer sequences. Supplementary Figure 1 MTT cell viability assay to assess DMSO safety. hDPCs were incubated with DMEM/2% FBS containing DMSO 0.1% or left untreated (hDPCs), and metabolic activity was evaluated daily during 6 days by MTT colorimetric assay. Supplementary Figure 2 Diagrams of experimental protocol for GW0742 treatment followed by inflammatory stimulus with LPS in hDPCs (2 μg/mL) and RAW264.7 cells (100 ng/mL), for (a) gene expression and gelatinolytic activity, and for (b) chemotaxis assay using coculture Transwell system. Supplementary Figure 3 (a) Treatment with 300 μM H2O2 significantly increases PPARβ/δ mRNA [file 8854921.f1.zip › Supp_Figure4.docx]

## Slide 1
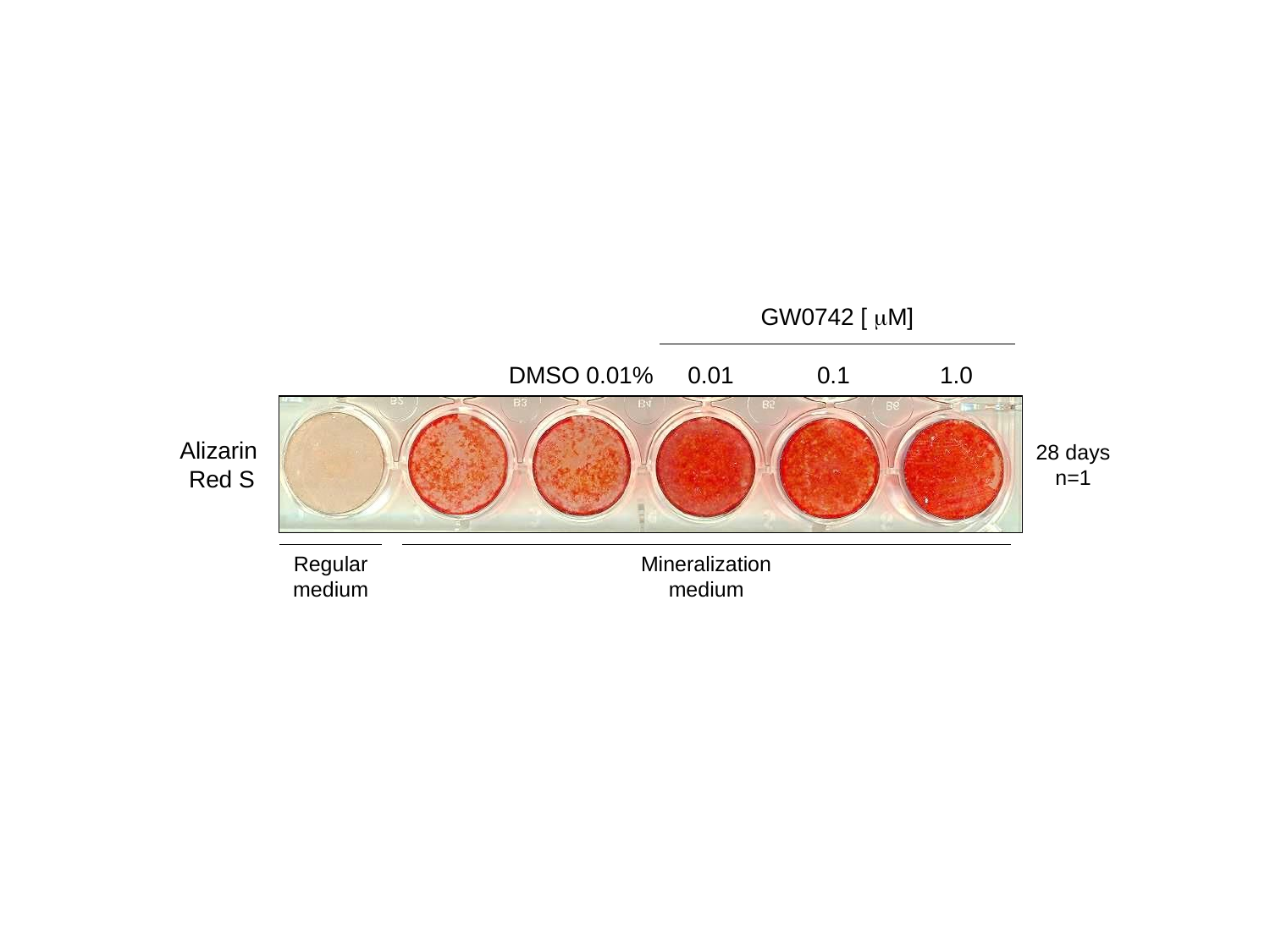

GW0742 [ mM]
DMSO 0.01%
0.01
0.1
1.0
Alizarin
Red S
28 days
n=1
Regular
medium
Mineralization
medium

Supplement: Supplementary Materials — Supplementary information may be found in the online version of this article: Supplementary methods. Quantitative real-time polymerase chain reaction: all quantitative real-time PCR assays were performed on the Applied Biosystems StepOnePlus™ Real-Time PCR Systems, and the data were generated by StepOne v2.1 Software. The cycle conditions were 50°C for 2 minutes, 95°C for 10 minutes, followed by 40 cycles at 95°C for 15 s and 60°C for 1 minute. Samples were normalized to beta-actin (hDPCs) or to glyceraldehyde 3-phosphate dehydrogenase enzyme (Gapdh; RAW264.7 cells). To assure specificity, a melt curve was obtained for all qPCR products. Further, standard curves were obtained for each primer pair to assess the efficiency of amplification. Mineralization assay: confluent hDPCs were cultured in mineralization medium (alpha minimum essential medium (α-MEM) with 10% FBS, antibiotics, 10 mmol/L β-glycerophosphate, 10 nmol/L dexamethasone, and 50 μg/mL ascorbic acid) containing GW0742 (1.0 μM) or vehicle (DMSO 0.01%). After 28 days, cells were rinsed with PBS, fixed with ethanol for 30 minutes at RT, and stained for 10 minutes with 2% Alizarin red S (Sigma-Aldrich) solution, pH 4.2, at RT. Cells were then rinsed 3 times with distilled water to reduce nonspecific staining. This experiment was performed in triplicate. Supplementary Table 1 Gene and primer sequences. Supplementary Figure 1 MTT cell viability assay to assess DMSO safety. hDPCs were incubated with DMEM/2% FBS containing DMSO 0.1% or left untreated (hDPCs), and metabolic activity was evaluated daily during 6 days by MTT colorimetric assay. Supplementary Figure 2 Diagrams of experimental protocol for GW0742 treatment followed by inflammatory stimulus with LPS in hDPCs (2 μg/mL) and RAW264.7 cells (100 ng/mL), for (a) gene expression and gelatinolytic activity, and for (b) chemotaxis assay using coculture Transwell system. Supplementary Figure 3 (a) Treatment with 300 μM H2O2 significantly increases PPARβ/δ mRNA [file 8854921.f1.zip › Supplementary_Figure_5 (1).pptx]
